# Supplementary figures and images for: Vitelline Warbler (Setophaga vitellina) songs, calls, and habitat preferences: novel acoustic descriptions of a range-restricted Caribbean songbird
Source: PLoS One. 2025 Apr 28;20(4):e0312636. doi: 10.1371/journal.pone.0312636 (PMC12036837; doi:10.1371/journal.pone.0312636)

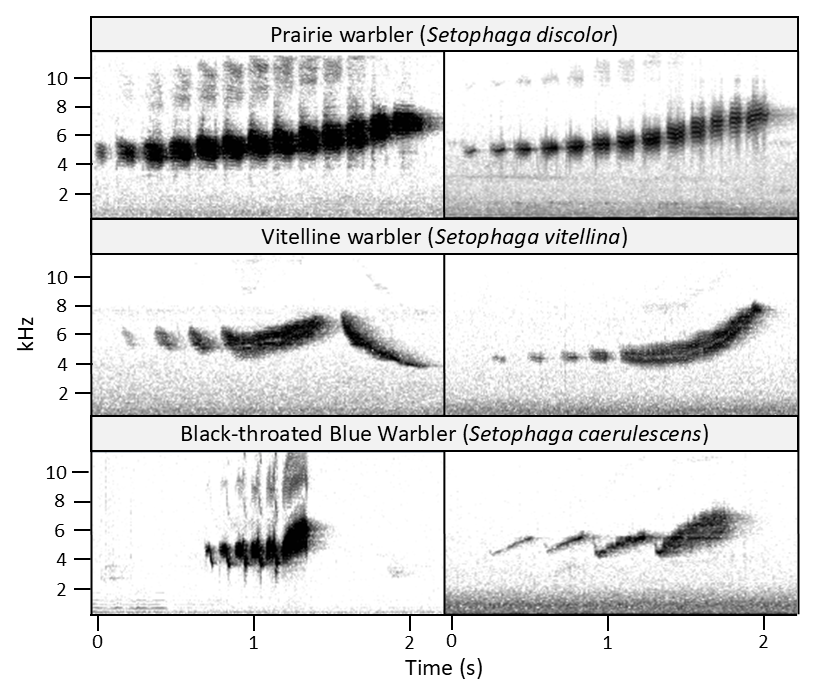

Supplement: S1 Fig — Both vitelline warbler songs are taken from our recordings, and the remaining four are used with permission from the Macaulay Library at the Cornell Lab of Ornithology: ML54975241 (Prairie Warbler, left), ML340590901 (Prairie Warbler, right), ML98819 (Black-throated Blue Warbler, left), ML616608500 (Black-throated Blue Warbler, right). (TIF) [file pone.0312636.s001.tif]
